# Severe fever with thrombocytopenia syndrome in children: a case report

**DOI:** 10.1186/1471-2334-14-366

**Published:** 2014-07-03

**Authors:** Li-Yuan Wang, Ning Cui, Qing-Bin Lu, Ying Wo, Hong-Yu Wang, Wei Liu, Wu-Chun Cao

**Affiliations:** 1 Graduate School of Anhui Medical University, Hefei 230032, P. R. China; 2 State Key Laboratory of Pathogen and Biosecurity, Beijing Institute of Microbiology and Epidemiology, Beijing 100071, P. R. China; 3 The 154 Hospital, People’s Liberation Army, Xinyang 464000, P. R. China; 4 School of Public Health, Peking University, Beijing 100191, P. R. China

**Keywords:** Severe fever with thrombocytopenia syndrome, Bunyavirus, Children

## Abstract

**Background:**

Severe fever with thrombocytopenia syndrome (SFTS) is an emerging infectious disease caused by a novel bunyavirus (SFTSV) in China. Humans of all ages living in endemic areas have high risk of acquiring SFTS. Most clinical data so far have been from adults and no clinical study was available from children yet. The present study identified four SFTSV infected children through hospital based surveillance. A prospective observational study was performed to obtain their clinical and laboratory characteristics.

**Case presentation:**

The patients’ age ranged from 4–15 years old and two were male. On hospitalization, fever, malaise and gastrointestinal syndromes were the most commonly presenting symptoms. Hemorrhagic symptoms or neurological manifestation was not recorded in any of the four pediatric patients. Hematological abnormalities at admission into hospital included leucopenia (4 cases), thrombocytopenia (1 case) and bicytopenia (1 case). The abnormal parameters included elevated aminotransferase (1 case), alanine transaminase (2 case), and lactate dehydrogenase (3 case). Laboratory parameters indicative of renal damage was not observed during the hospitalization. All the patients recovered well without sequelae being observed.

**Conclusion:**

Compared with adults, pediatric patients with SFTSV infection seem to have less vague subjective complaints and less aggressive clinical course. Thrombocytopenia is suggested to be used less rigorously in recognizing SFTSV infection in pediatric patients, especially at early phase of disease.

## Background

Severe fever with thrombocytopenia syndrome (SFTS) is an emerging infectious disease caused by a novel bunyavirus (SFTSV) that was firstly described in 2010 in China [1], Outside China, confirmed SFTS or SFTS like patients had recently been reported in Korea, Japan, Dubai, United Arab Emirates and Missouri, United States, suggesting its expanded distribution in other countries [2-5] The disease usually presents as fever, thrombocytopenia and leukocytopenia, with case-fatality rates ranging from 2.5% to 30% [6]. Epidemiological investigation revealed that SFTS cases mostly lived in rural areas working as farmers, and their age ranged from 1 to 90, with most part of cases (75%) older than 50 years old [1,5,6]. Although pediatric patients under 18 years old were recorded, most data available so far have been from adults and no clinical study on children was performed yet.

Since 2011, we have performed a hospital based surveillance on SFTSV infected cases in one SFTS-designated hospital in Xinyang, Henan Province, China [7]. From the same hospital, we identified four pediatric cases confirmed to be infected with SFTSV by real-time reverse transcriptase polymerase chain reaction (RT-PCR) and subsequent sequencing [7]. A prospective observational study was performed to obtain their clinical and laboratory characteristics from acute phase of infection until convalescence. Altogether 180 SFTSV infected adult patients who had been hospitalized during the same period from the same hospital were extracted for comparison with these pediatric patients.

## Case presentation

The median age of the pediatric patients was 8 (range 4–15) years old and 2 were male. Two of the patients had tick bite history during their outdoor activity. The other two had close contacts with their SFTSV infected family members, which is a higher frequency than in adults (6.7%). All the pediatric patients were otherwise healthy and had no previous co-morbidity. The delay from disease onset to hospital entrance was 3–7 days, comparable with that of adults (mean 5 days). On hospitalization, fever, malaise and gastrointestinal syndromes were the most commonly presenting symptoms. This is similar with the results that were obtained from adult patients (Table 1). No syndromes of dyspnea, headache, consciousness disorder, dizziness, sputum production, cough or myalgias were observed from the pediatric patients, which were presented with frequencies ranging from 11.7% to 82.2% in adult patients. Hemorrhagic symptoms (hematuria, petechia, hematemesis, gingival bleeding, melena), which were manifested in adult patients were rarely found in these pediatric patients, except for the presence of petechia in case 1. No neurological manifestation was recorded in any of the four pediatric patients.

**Table 1:** Demographic and clinical characteristics of the children with SFTS in comparison with adult patients

**Characteristics**	**Pediatric patients**				**Adult patients (N = 180)**
	**Case1**	**Case2**	**Case3**	**Case4**	
**Demographic features**					**No. (%)/mean (SD)**
Gender	Male	Female	Female	Male	80 male (44.4)
Age, y,	6	7	4	15	57.5 (13.0)
History of tick bite	Yes	No	No	Yes	29 (16.1)
Close contact with known SFTS patients	No	Yes	Yes	No	12 (6.7)
					**Mean (range)**
Median days from syndromes to admission	3	7	5	4	5 (1–38)
**Presenting with clinical manifestations on admission**					**No. (%) of patients**
Fever	Yes	Yes	Yes	Yes	177 (98.3)
Malaise	Yes	Yes	Yes	Yes	172 (95.6)
Myalgias	No	No	No	No	148 (82.2)
Gastrointestinal syndromes	Yes	Yes	Yes	No	116 (64.4)
Cough	No	No	No	No	63 (35.0)
Sputum production	No	No	No	No	46 (25.6)
Dizziness	No	No	No	No	42 (23.3)
Headache	No	No	No	No	35 (19.4)
Dyspnea	No	No	No	No	21 (11.7)
Arthralgias	No	Yes	No	No	13 (7.2)
Lymphadenopathy	No	Yes	No	No	78 (3.3)
Hematuria	No	No	No	No	33 (18.3)
Petechia	No	No	Yes	No	25 (13.9)
Hematemesis	No	No	No	No	13 (7.2)
Gingival bleeding	No	No	No	No	2 (1.1)
Melena	No	No	No	No	2 (1.1)
Consciousness disorder	No	No	No	No	36 (20.0)
**Laboratory measurements on admission**					**Mean ± SD**
WBC (x10^9^/L)	2.3*	1.83*	2.9*	3.3*	3.0 ± 2.3
PLT (x10^9^/L)	118	87*	107	148	71.3 ± 34.6
Neutrophils (%)	52.9	41.7	37.3	72.5	62.0 ± 15.9
Lymphocyte (%)	32.7	43.7	58.1	19.2	29.1 ± 13.0
**With abnormal laboratory tests on admission**					**No. (%) of patients**
AST >40 U/L	No (14)	Yes (521)	No (18)	No (39)	148 (82.2)
ALT >40 U/L	No (32)	Yes (178)	Yes (55)	No (19)	115 (63.9)
ALB <35 g/	No (48.3)	No (42.5)	No (45.5)	No (44.4)	78 (45.6)
ALP >150 U/L	Yes (289)	Yes (239)	Yes (191)	No (132)	14 (8.2)
GGT >50 U/L	No (10)	Yes (52)	No (13)	No (15)	49 (27.2)
LDH >245 U/L	Yes (250)	Yes (545)	Yes (320)	No (236)	136 (75.6)
CK >232 U/L	No (89)	Yes (1167)	No (94)	Yes (1012)	128 (71.1)
BUN >7.8 mmol/L	No (5.68)	No (3.36)	No (4.59)	No (7.5)	45 (25.0)
CREA >97 mmol/L	No (30)	No (35)	No (35)	No (87)	41 (22.8)
Proteinuria	No	No	No	No	55 (30.6)
**Outcome**					
Recovery	Yes	Yes	Yes	Yes	144 (80.0)
Adverse Outcome	No	No	No	No	36 (20.0)
Death	No	No	No	No	25 (13.9)

*Denotes abnormal value.

*Abbreviations*: *WBC* white blood cell, *PLT* platelet, *HGB* hemoglobin, *AST* aspartate aminotransferase, *ALT* alanine transaminase, *ALB* albumin, *ALP* alkaline phosphatase, *GGT* gamma-glutamyl transpeptidase, *LDH* lactate dehydrogenase, *CK* creatine kinase, *BUN* blood urea nitrogen, *CREA* Creatinine.

For the laboratory tests, hematological abnormalities at admission into hospital included leucopenia (4 cases), thrombocytopenia (1 case) and bicytopenia (1 case). The abnormal parameters indicative of liver damage, for example, aspartate aminotransferase (AST), alanine transaminase (ALT), and lactate dehydrogenase (LDH) were presented in one, two and three patients, respectively. The frequencies in adults ranged from 63.9% (ALT) to 82.2% (AST). None of the pediatric patients had elevated albumin (ALB), blood urea nitrogen (BUN), creatinine (CREA), or presence of proteinuriay, which were mostly indicative of renal damage. These abnormalities were present in 45.6%, 25%, 22.8% and 30.6% among adult patients, respectively. At the end of clinical course, all patients presented different degree of neutropenia.

**Figure 1:**
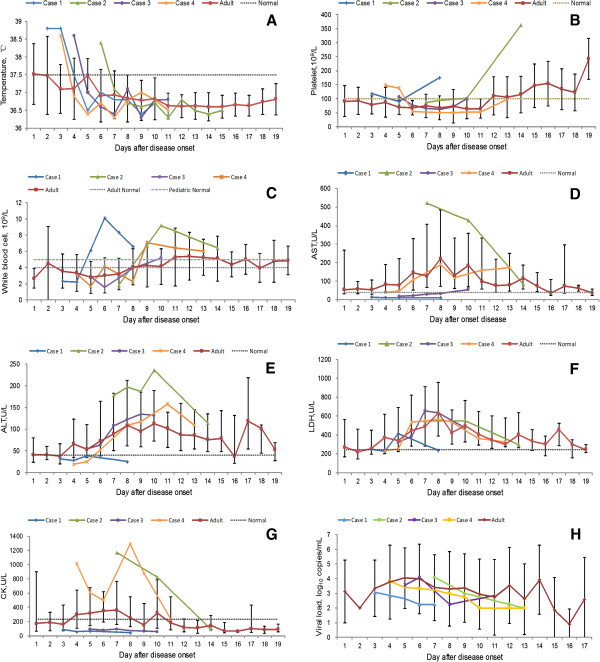
**Dynamic profiles of laboratory parameters in 4 SFTS pediatric patients in comparison with 180 adult patients.** The laboratory parameters include temperature **(A)**, platelet counts **(B)**, white blood cell counts **(C)**, aspartate aminotransferase (AST) **(D)**, alanine transaminase (ALT) **(E)**, lactate dehydrogenase (LDH) **(F)**, creatine kinase (CK) **(G)** and SFTSV viral loads **(H)**. Dynamic profiles were delineated using mean (standard deviations) in **A**, **B**, **C** and **H** and median (P25-P75) in **D**, **E**, **F** and **G**. The lowest platelet counts (x10^9^/L) in Figure 1**B** were 90,87, 62 and 50, respectively. The lowest WBC counts (x10^9^/L) in Figure 1**C** were 2.2, 1.8, 1.6 and 1.7, respectively. The highest values of AST (U/L) in Figure 1**D** were 14, 521, 54 and 188, respectively. The highest levels of ALT (U/L) in Figure 1**E** were 39, 236, 132 and 158, respectively. The normal ranges of the laboratory parameters were delineated for children and adults separately. Only one range was marked if the normal ranges were the same for children and adults.

The pediatric patients had peak temperature of 38.8°C after hospitalization, declining to normal level at 4–8 days post disease onset. In contrast, the estimated median fever course in adult patients was 2 days (Figure 1A). The dynamic evaluations of PLT revealed a delayed occurrence of thrombocytopenia in three patients (5–6 days after disease onset) than that of the adults (mean 1–2 days). In addition, the recovery of PLT to normal levels was earlier than that of the adults (5–9 *vs* 10 days). The nadir levels of WBC counts in all the 4 children were lower than the mean level of adults at the same time points post infection, yet with a more rapid recovery than that of adults in case 1, 2 and 4 (Figure 1C).For the other four abnormal indicators, AST and CK were maintained at significantly lower level than the adults (Figure 1C and G), and case 1 additionally had lower level of ALT (Figure 1E). Similar patterns were observed between pediatric and adult patients for other evaluations (Figure 1D-G). It’s noteworthy that for case 3 and case 4, ALT and AST levels were normal on admission, only elevated to abnormal during the follow-up period. During the whole hospitalization, all the pediatric patients had normal BUN, CREA and ALB levels and no hemorrhagic symptoms other than petechia developed (data not shown).

At 2-month revisit to the hospital, the patients remained healthy without sequelae. patient 2 and patient 3 developed antibody titers of 1:160 and 1:640 by enzyme-linked immunosorbent assay (ELISA) using the recombinant nucleoprotein of SFTSV [7]. In comparison, the IgG antibody titers of their household members from whom the infection was acquired were both 1:320. The other two pediatric patients failed to be revisited on the 2-month revisit.

## Discussion and conclusions

In the current study, no death occurred among 4 pediatric patients and their clinical manifestation appeared to be milder than those of the adult patients. Apart from fever and gastrointestinal syndromes, the pediatric patients presented with less vague subjective complaints, as well as rare hemorrhagic symptom which were more often presented in adult patients. This made the recognition of pediatric SFTS cases more difficult than the adults, solely based on clinical syndromes. According to the original diagnosis criteria, a clinical SFTS patient was defined as presenting both thrombocytopenia and lymphopenia [8]. However, thrombocytopenia is not evident until several days after hospitalization according to the current finding from pediatric patients. These patients might be missed if diagnosed by the standard criteria. We therefore propose that thrombocytopenia should be used less rigorously in recognizing SFTSV infection in pediatric patients at early phase of disease.

In clinical practice, the laboratory parameters indicative of liver and renal damage were regularly used for auxiliary diagnosis of SFTS. As displayed in the current pediatric patients, laboratory parameters indicative of liver damage (AST, ALT, LDH, CK), instead of renal damage (BUN, CREA and ALB) was manifested. The evaluation of AST, ALT, LDH and CK could play roles in the auxiliary diagnosis; however they need to be used cautiously, as these evaluations might not be elevated at early infection and also kept at normal levels in certain pediatrics during the whole hospitalization.

Disease severity of patients infected with SFTSV varied, depending on multiple factors, including the virulence of different strains, access to the health system, the host factors, as well as other unknown reasons. Since the current pediatric patients acquired infection from the same region during the same period with adult patients, the impact from infected viral strain was supposed to be minor. Moreover, the children have better access to the health system. The comparably milder disease in pediatric patients therefore might be derived from the host factor. Jin et al. have demonstrated that virus replication and overexuberant immune responses can contribute to progressive organ damage [9]. Moreover, dramatically elevated proinflammatory cytokine levels have been detected in severe or fatally infected patients, suggestive of a cytokine storm might contribute to the adverse disease outcome [10-12]. Children are physically different with adults and the incomplete maturation of the immune system could pose as one possible mechanism underlying their milder disease manifestation in comparison to adult patients [13]. This mechanism had been applied in explaining the similarly milder clinical course of Crimean–Congo hemorrhagic fever (CCHF) in children than the adults [13].

The current study is subject to major limitation that only observation study was made on four patients without statistical analysis performed to draw validated conclusion. This needs to be addressed by study of large sample size in the future.

### Ethical approval

The research protocol was approved by the Human Ethics Committee of the 154 Hospital (No. 003/51). All guardians of the participants provided written informed consent.

### Consent

Written informed consent was obtained from the patient for publication of this Case report and any accompanying images. A copy of the written consent is available for review by the Editor of this journal.

## Abbreviations

SFTS: Severe fever with thrombocytopenia syndrome; WBC: White blood cell; AST: Aminotransferase (AST); ALT: Alanine transaminase; LDH: Lactate dehydrogenase; ALB: Albumin; BUN: Blood urea nitrogen; CREA: Creatinine.

## Competing interests

The authors declare that they have no competing interests.

## Authors’ contributions

WCC and WL conceptualized and designed the study and approved the final manuscript as submitted. LYW and YW carried out the experiments and drafted and reviewed the manuscript, and approved the final manuscript as submitted. NC, QBL and HYW collected the samples and information of the patients, analyzed the data and made the figures, reviewed and approved the manuscript.

## Pre-publication history

The pre-publication history for this paper can be accessed here:

http://www.biomedcentral.com/1471-2334/14/366/prepub
